# Unsupported MoS_2_-Based Catalysts for Bio-Oil Hydrodeoxygenation: Recent Advances and Future Perspectives

**DOI:** 10.3389/fchem.2022.928806

**Published:** 2022-06-17

**Authors:** Jing Cao, Youming Zhang, Li Wang, Cen Zhang, Congshan Zhou

**Affiliations:** Department of Chemistry and Chemical Engineering, Hunan Institute of Science and Technology, Yueyang, China

**Keywords:** bio-oil, hydrodeoxygenation, unsupported MoS_2_, morphology, defect, metal doping, deactivation

## Abstract

In recent years, unsupported MoS_2_-based catalysts have been reported as promising candidates in the hydrodeoxygenation (HDO) of bio-oil. However, preparing MoS_2_-based catalysts with both high activity and good stability for HDO reaction is still challenging and of great importance. Hence, this mini-review is focused on the recent development of unsupported MoS_2_-based HDO catalysts from the understanding of catalyst design. The three aspects including morphology and defect engineering, metal doping, and deactivation mechanism are highlighted in adjusting the HDO performance of MoS_2_-based catalysts. Finally, the key challenges and future perspectives about how to design efficient catalysts are also summarized in the conclusions.

## Introduction

With the continuous consumption of fossil fuels, the energy problem has become more serious in modern society. Therefore, a lot of research studies in the energy field have focused on finding a new fuel that can replace traditional fossil fuels, which could be compatible with existing infrastructure, sustainable, and reduce CO_2_ emissions (Jiang et al., 2021). In recent years, bio-oil obtained from flash pyrolysis of biomass is considered a potential fuel in the future because of its wide range of raw materials and renewable (Qu et al., 2021).

**FIGURE 1:**
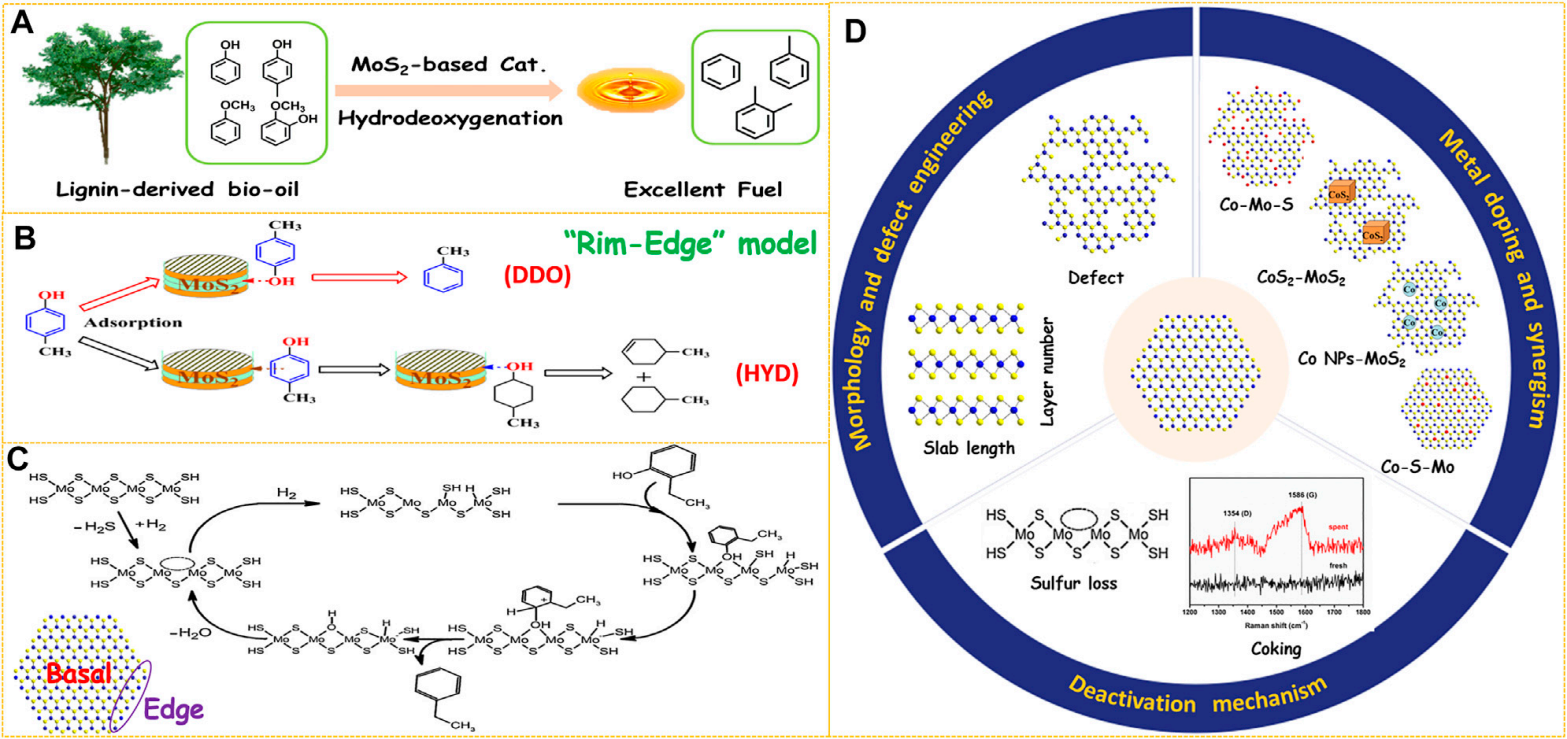
**(A)** Upgrading of lignin-derived bio-oil through HDO process. **(B)** Reaction pathways over MoS_2_-based catalysts. Reproduced with permission from (Wang et al., 2014d). Copyright (2014) American Chemical Society. **(C)** Proposed HDO mechanism of 2-ethylphenol on MoS_2_-based catalyst. Reproduced with permission from (Mortensen et al., 2011). Copyright (2011) Elsevier. **(D)** Three aspects to improve the HDO performance of MoS_2_-based catalysts.

Compared with fossil fuels, the oxygen content in bio-oil is very high (up to 40 wt.%). This component characteristic results in the disadvantages of low heating value, high viscosity and acidity, and poor thermal stability. Therefore, bio-oil must be upgraded through hydrodeoxygenation (HDO) to reduce the oxygen content before being used as an excellent fuel (Figure 1A). Thus, developing catalysts with excellent catalytic performance is crucial for the bio-oil HDO process. As the most commonly used catalysts for hydrodesulfurization (HDS) in industry, supported transition metal sulfides (CoMoS/Al_2_O_3_ or NiMoS/Al_2_O_3_) also have been frequently investigated in the HDO reaction due to their low cost and mature technology. However, the HDO activity of supported catalysts is not very ideal, which usually requires harsh reaction conditions of 300°C or above. Under high reaction temperature, the sulfide catalysts will undergo a fast deactivation due to sulfur loss without sulfur compensation from external sources during the reaction process (Wandas et al., 1996; Wang et al., 2015a; Liu et al., 2017). Recently, unsupported MoS_2_-based catalysts have been reported to show much better HDS and HDO activity than supported counterparts because of the high density of active sites, which is especially suitable for dealing with large molecules (Wang et al., 2009; Wang et al., 2014b). More importantly, the unsupported catalysts provide an ideal platform for studying the structure-performance relationship due to the absence of support. Over the past decade, a lot of studies have been carried out on how to improve the HDO activity, selectivity, and stability of unsupported MoS_2_-based catalysts.

Hence, in this mini-review, we firstly reviewed in detail how to study the structure-performance relationship through morphology and defect engineering of MoS_2_ catalysts. Then, the effect of metal doping on the active phase structure and HDO performance of the MoS_2_ catalyst are summarized. Moreover, the deactivation mechanism and how to inhibit the deactivation of MoS_2_-based catalysts in the HDO process is also introduced. Finally, we analyzed the challenges and future opportunities to design highly efficient unsupported MoS_2_-based HDO catalysts.

## Morphology and Defect Engineering

MoS_2_ is a typical two-dimensional layered compound, which has two morphology parameters of slab length and layer number (Figure 1D). These two parameters could be visualized by high-resolution transmission electron microscopy (HRTEM) observation from the length and number of black fringes in the HRTEM images, respectively (Lai et al., 2016; Cao et al., 2021b). For unsupported MoS_2_-based catalysts, it is important to boost the number of active sites by increasing the Mo dispersion. Hence, shortening the slab length or decreasing the layer number of MoS_2_ can both improve its HDO activity. Due to the weak Van der Waals force between the adjacent layers, MoS_2_ can be easily exfoliated into few-layer or even single-layer nanosheets by some physical or chemical methods (Zhu et al., 2018; Cao et al., 2021c). A simple and effective hydrazine-assisted liquid exfoliation method was developed to exfoliate the commercial MoS_2_ to few-layer nanosheets (Liu et al., 2016). The HDO activity was remarkably enhanced for few-layer MoS_2_ nanosheets due to the exposure of more active surface sites. In addition, the layer number of bulk MoS_2_ was further decreased to monolayer by *n*-butyllithium exfoliation, and the conversion was extraordinarily improved from 25.8% of bulk MoS_2_ to 98.7% of single-layer MoS_2_ in the HDO of 4-methylphenol (Liu et al., 2017). This result demonstrates that preparing few-layer, especially monolayer MoS_2_ is an effective way to design highly efficient HDO catalysts. Various methods have been developed to synthesize unsupported MoS_2_ catalysts such as hydrothermal (Cao et al., 2021a; Zhang C. et al., 2021), ball milling (Wang C. et al., 2014), thermal decomposition (Yi et al., 2011), and solution method (Genuit et al., 2005). Among the abovementioned methods, the hydrothermal method is most widely used due to the advantages of simple operation and controllable morphology of the product. In the hydrothermal procedure, temperature, pH value, and surfactants showed a great effect on the morphology of the MoS_2_ product. For example, an acidic environment in the hydrothermal process was helpful to facilitate the nucleation and prepare smaller MoS_2_ particles, thus resulting in enhanced activity in the HDO of *p*-cresol (Wang et al., 2015b; Zhang C. et al., 2018). A higher initial synthesis temperature could promote fast nucleation and shorten the MoS_2_ slab length (Zhang et al., 2015). The layer number of MoS_2_ was adjusted successfully by adding different types of surfactants in the hydrothermal procedure and then the morphology-performance relation in the HDO of 4-methylphenol was studied (Wang et al., 2014d). There are two parallel reaction routes for the HDO of phenols over MoS_2_-based catalysts, which are defined as direct deoxygenation (DDO) and hydrogenation-dehydration (HYD). It was found that MoS_2_ catalyst with lower stacking showed a higher HYD selectivity, and the DDO route was favored by using MoS_2_ with higher stacking degree (Figure 1B). This phenomenon was well explained by the Rim-Edge model proposed in the HDS field (Daage and Chianelli, 1994). Therefore, the HDO selectivity could be precisely regulated by adjusting the layer number of MoS_2_ particles.

Generally, the coordinatively unsaturated sites (CUS) on the edge planes are considered active centers of MoS_2_ catalysts while the atoms on the basal planes are catalytically inert (Figure 1C) (Lauritsen et al., 2004). Therefore, creating defects on the basal planes is another important approach to increase the number of active sites (Figure 1D). Some strategies such as etching (Zhang W. et al., 2018; Wang et al., 2020), gas treatment (Li et al., 2019), or plasma bombardment (Tao et al., 2015) have been utilized to create defect sites on the basal planes. The location and type of defects can be directly observed by HRTEM, and the quantity information can be obtained by electron paramagnetic resonance (EPR) characterization (Wu et al., 2020). Recently, we have synthesized MoS_2_ nanosheets with rich defects through *in situ* etching by adding excess sulfur sources *via* a simple one-pot hydrothermal method (Zhang C. et al., 2021). The HDO activity was improved 1.7 folds through defect engineering within the basal planes. Zhang et al. reported a facile H_2_O_2_ etching strategy to tailor the concentration of sulfur vacancies by altering the H_2_O_2_/MoS_2_ molar ratio (Zhang Y. et al., 2021). The catalytic performance evaluation results revealed a linear relationship between the HDO activity and the degree of sulfur vacancies, further suggesting that sulfur vacancies acted as catalytic centers for the HDO reaction. The synthesis of amorphous MoS_2_ with low crystallinity is also one of the means to increase the number of defects. It was found that incorporating organic solvents or hydrazine in the hydrothermal method was conducive to obtaining amorphous MoS_2_ with a defect-rich structure. A bent and multilayered amorphous MoS_2_ was synthesized by utilizing (NH_4_)_2_MoS_4_ as a raw material and decalin as an organic solvent, and the HDO activity was enhanced by 31% compared with the crystalline MoS_2_(Yoosuk et al., 2012). Co-doped MoS_2_ nanoflowers with abundant defects by adding hydrazine to the hydrothermal system was prepared, which displayed an excellent p-cresol HDO performance (Song et al., 2018a). More importantly, the defects on the basal planes could provide additional anchor sites to accommodate promoter atoms. Recently, we have found that the content of the Co-Mo-S active phase was improved significantly by creating numerous defects on the basal planes of MoS_2_ support even impregnated with the same amount of Co promoters (Zhang C. et al., 2021). As a result, the Co-doped few-layer and defect-rich MoS_2_ achieved a 100% HDO conversion of p-cresol and 99.7% selectivity of toluene at 230°C. The increased surface sulfur defects produced by H_2_O_2_ etching were ideal platforms for stabilizing Co atoms to form the Co-Mo-S phase. The HDO activity of Co-MoS_2_ catalysts was increased 3.4 times by optimizing the etching conditions (Zhang Y. et al., 2021).

## Metal Doping and Synergism Study

In general, the activity of unpromoted MoS_2_ is far from sufficient for catalyzing the HDO reaction effectively. Hence, it is essential to weaken the Mo-S bond through metal doping to reduce the formation temperature of sulfur vacancy. Co is the most commonly used metal to promote MoS_2_ because the DDO route is more favored for Co-doped catalysts which could minimize the H_2_ consumption. Based on the reported literature, there are mainly four different structures of catalytic centers for Co-promoted MoS_2_ catalysts (Figure 1D): (I) Co-Mo-S, (II) CoS_2_-MoS_2_, (III) Co-S-Mo (or called Co atom-MoS_2_), and (IV) Co NPs-MoS_2_ (NPs: nanoparticles). The four different catalytic active phases were obtained by tailored preparation methods, which resulted in a variety of synergistic mechanisms.

The Co-Mo-S model is the most accepted active phase structure in the field of HDS, in which Co atoms are preferentially located on the edge planes of MoS_2_. In our recent work, Co-doped nano-MoS_2_ catalysts with Co-Mo-S as an active phase were obtained by impregnating the as-prepared MoS_2_ with Co(CH_3_COO)_2_ followed by sulfidation in H_2_S/H_2_ atmosphere (Cao et al., 2021a). The temperature-programmed reduction (TPR) results displayed that the reduction peak was shifted from 285°C for MoS_2_ to 224°C for Co-doped nano-MoS_2_, which suggests a greatly decreased temperature for producing coordinative unsaturated sites (CUS). Also, it was found the Co content had a huge effect on the structure of the active phase and catalytic performance. If a small amount of Co are introduced, all Co atoms are preferentially located at the edge planes to form the Co-Mo-S phase. When the content of Co is further increased, the Co_9_S_8_ phase is gradually formed after the edge sites are fully occupied. However, too much Co content is not suggested because the larger Co_9_S_8_ would block some of the active sites. Also, the optimized catalyst with a Co/(Co + Mo) molar ratio of 0.3 had both Co-Mo-S and small Co_9_S_8_ particles, which embodies a synergistic effect between Co-Mo-S and Co_9_S_8_ phases. The HDO activity and toluene selectivity were improved dramatically from 16.9 to 65.0% of MoS_2_ to 98.7 and 98.9% of Co/MoS_2_-0.3 at 220°C. Another well-known model in the field of hydrorefining is the Remote-Control model, in which the independent Co_*x*_S_*y*_ phase acts the role to provide spillover hydrogen. Wang et al. adopted a two-step hydrothermal method which obtained separated CoS_2_ and MoS_2_ phases in the resulted catalysts rather than the Co-Mo-S phase prepared by one-step hydrothermal method (Wang et al., 2016b). The CoS_2_/MoS_2_ showed an excellent HDO activity of 97.8% and a high toluene selectivity of 99.2% at 250°C. The unprecedented HDO performance was attributed to the large surface area and the synergism between CoS_2_ and MoS_2_. More interestingly, the CoS_2_ and MoS_2_ interface was *in situ* reconstructed into Co-Mo-S by H_2_ pre-reduction treatment, resulting in a large amount of surface Co-Mo-S active phase toward efficient HDO of p-cresol (Liu et al., 2020). In early electrochemical research, researchers found that sulfur vacancy has a strong adsorption capacity for Co complexes. On the basis of this discovery, Liu et al. prepared a single-layer MoS_2_ catalyst doped with isolated Co atoms (Co-^S^MoS_2_) by hydrothermal treatment of monolayer MoS_2_ with Co(thiourea)_4_^2+^. The EXAFS and HAADF-STEM characterization results confirmed that single Co atoms were covalently bonded to the sulfur vacancies on the basal planes to form a new Co-S-Mo active phase (Liu et al., 2017). The theoretical calculation results showed that the proximal sites around Co-S-Mo were energetically favorable for the formation of sulfur vacancies, thus making Co-^S^MoS_2_ an extremely active and selective catalyst in the HDO reactions. Meanwhile, superior stability was also observed due to the low operating temperature at 180°C. An *in situ* interfacial reactions was adopted to prepare MoS_2-x_ catalyst supported by Co nanoparticles (Co NPs-MoS_2_) based on the reducibility of defection-rich MoS_2-x_ (Wu et al., 2020). The HRTEM and DFT results showed that Co NPs were energetically favored to adsorb on the defects to form a metal-vacancy interface. Also, the Co NPs promoted the formation of new sulfur vacancies at the proximal sites as evidenced by TPR results. More importantly, the DFT results showed that the adsorption energy of 4-methylphenol and the reaction energy barrier were significantly decreased on Co NPs-MoS_2_, which endowed the catalyst exhibit the lowest reaction temperature (120°C) up to now.

Ni is also a commonly used promoter to improve the catalytic performance of MoS_2_. However, Ni-promoted MoS_2_ catalysts usually have high hydrogenation activity because Ni is more capable of adsorbing and activating H_2_. Ni-Mo-S and NiS_2_//MoS_2_ catalysts with the same Ni/Mo molar ratio by one-step hydrothermal method and two-step hydrothermal method were prepared, respectively (Wang et al., 2015a). It was found that NiS_2_//MoS_2_ showed a much higher p-cresol conversion than Ni-Mo-S, which indicated that the synergistic effect between Ni promoters and MoS_2_ came from the spillover hydrogen rather than the Ni-Mo-S phase (Wang et al., 2016a). The optimized NiS_2_//MoS_2_ showed a p-cresol conversion of 95.8% and a methylcyclohexane selectivity of 55.6% at 275°C for 4 h. Although Fe is much cheaper than Co and Ni, Fe has attracted little research interest because of its poor performance in promoting MoS_2_. Recently, Fe-promoted MoS_2_ catalysts by one-step hydrothermal method, which exhibited separated FeS_2_ and MoS_2_ phases were prepared (Guo et al., 2019). However, the FeS_2_ was transformed into FeS after a pretreatment in H_2_ and the HDO activity was significantly enhanced. The authors concluded that FeS was a better promoter than FeS_2_ for MoS_2_, and acts as a donor of activated hydrogen in the HDO reaction.

According to a recent report, precious metals such as Pt can also play a synergistic role in promoting the HDO activity of unsupported MoS_2_ catalysts. A metal insertion-deinsertion strategy was adopted to prepare Pt-MoS_2-x_ catalysts in which Pt^4+^ initially substituted the Mo^4+^ on the basal planes and then Pt species was deinserted to be anchored on the adjacent sites (Wu et al., 2022). As a result, a large number of edge sites were created at the proximal sites of the Pt-edge interface within the basal planes which are originally inert. Thus, the optimized Pt-MoS_2-x_ catalyst displayed extraordinary catalytic activity in the HDO of p-cresol, which achieved a conversion of 100% and a methylcyclohexane selectivity of 96.3% at 120°C. The DFT calculation results showed that the metal-edge interface was beneficial for lowering both the adsorption energy of reactants and the energy barrier of the HDO reaction. We have summarized the HDO performance of some unsupported MoS_2_-based catalysts in the past decade utilizing p-cresol as a probe molecule (Table 1).

**TABLE 1:** Comparison of the catalytic performance in the HDO of *p*-cresol for MoS_2_-based catalysts from the literatures.

Entry	Catalyst	Conditions					Catalytic performance			Ref.
		T (^o^C)	P (MPa)	t (h)	Catalyst (g)	Reactor type	*p*-cresol (mmol)	Conv. (%)	Toluene Select. (%)	
1	Co/MoS_2_-FL-DR	230	3.0	\	0.5	Continuous	2.3	100	99.7	Zhang et al. (2021a)
2	Co/nano-MoS_2_	220	3.0	\	0.5	Continuous	2.3	98.7	98.9	Cao et al. (2021a)
3	Co-Mo-S	275	4.0	4	0.3	Batch	138.9	100	92.2	Wang et al. (2014b)
4	CoS_2_/MoS_2_	250	4.0	1	0.03	Batch	44.4	98	99	Wang et al. (2016b)
5	Flower-like Co-doped MoS_2_	300	4.0	4	0.02	Batch	5.2	99.8	97.9	Song et al. (2018a)
6	CoMoS	300	4.0	3	0.02	Batch	5.0	100	100	Song et al. (2018b)
7	Flower-like Co-Mo-S	275	4.0	5	0.1	Batch	133.3	85.6	97.5	Wang et al. (2016c)
8	CoMo/Al_2_O_3_	360	7.0	1	1.0	Batch	4.0	95	18	Wandas et al. (1996)
9	CoMoS	300	3.0	6	0.01	Batch	1.3	99.1	94.4	Liu et al. (2020)
10	Co-^S^MoS_2_	180	3.0	8	0.02	Batch	2.6	97.6	98.4	Liu et al. (2017)
11	Co-MoS_2-*x*_	120	3.0	6	0.04	Batch	5.2	97.4	99.5	Wu et al. (2020)
12	Pt-MoS_2-*x*_	120	5.0	10	0.2	Batch	2.4	100	3.7	Wu et al. (2022)
13	Ni-Mo-S	300	4.0	6	0.6	Batch	125	99.9	28.8	Wang et al. (2015a)
14	NiS_2_//MoS_2_	275	4.0	4	0.3	Batch	125	95.8	35.6	Wang et al. (2016a)
15	NiMoWS	300	3.0	5	0.2	Batch	25.6	97.8	87.2	Wang et al. (2014a)
16	Ni-Mo-W-S	300	4.0	6	0.2	Batch	41.7	97.9	30.1	Wang et al. (2014c)
17	Fe/MoS_2_	250	4.0	3	0.03	Batch	29.6	96.3	94.5	Guo et al. (2019)

## Deactivation Mechanism and Measures to Inhibit Deactivation

Deactivation is a serious problem when MoS_2_-based catalysts are applied in the HDO reactions, which include two main causes such as sulfur loss and coking (Figure 1D). As the bio-oil does not contain sulfur, sulfur will be gradually lost due to S-O exchange, and the presence of H_2_O product will aggravate this process. Therefore, the following strategies have been carried out in order to inhibit catalyst deactivation due to sulfur loss. The first type of method is the co-feeding of sulfur-containing compounds such as H_2_S or benzothiophene. Adding an appropriate amount of H_2_S to the reaction system could well maintain the activity and stability of NiMo and CoMo catalysts due to the increased concentration of -SH groups (Dabros et al., 2018). A small amount of benzothiophene in the feed is helpful to inhibit catalyst deactivation because the H_2_S molecules produced by HDS of benzothiophene will adsorb on the active sites and weaken the negative effect of water (Wang et al., 2018). However, excessive benzothiophene introduction resulted in a decrease in HDO activity due to competitive adsorption between H_2_S and p-cresol. More importantly, it should be noted that contamination of bio-oil products is inevitable by adding sulfiding agents. To overcome this shortcoming, a surface hydrophobic treatment strategy was proposed in which the generated water is removed from the surface immediately, thus improving the cyclic stability of MoS_2_-based HDO catalysts. For example, the surface hydrophobicity of MoS_2_ was enhanced significantly by silicomolybdic acid modification as revealed by the contact angle results (Wu et al., 2019). After recycling for three runs, the p-cresol conversion of the modified MoS_2_ catalyst only showed a slight loss of 0.4%, which is much lower than that of the original MoS_2_ (9.2%). Carbon coating is another surface hydrophobic modification method to prevent sulfur loss in the HDO process. Amorphous carbon-coated CoS_2_-MoS_2_ catalysts were prepared by adding polyvinylpyrrolidone (PVP) to the hydrothermal system (Wang et al., 2017). The hydrophobic carbon layer covered on the surface effectively prevented the oxidation caused by water, and the HDO activity of CoS_2_-MoS_2_ decreased by only 2.6% after recycling three times. Lowering the reaction temperature is also an effective way to inhibit sulfur loss. The Co-^S^MoS_2_ maintained excellent HDO activity and selectivity without sulfur loss after using seven cycles, which is attributed to the greatly decreased reaction temperature from 300°C of traditional CoMo/Al_2_O_3_ catalysts to 180°C (Liu et al., 2017). The Co NPs-MoS_2_ and Pt-MoS_2-x_ catalysts reported (Wu et al., 2020; Wu et al., 2022) also showed superior stability in the HDO of p-cresol due to the further lowered operating temperature of 120°C.

Coking is a general problem for catalyst deactivation by decreasing the surface area and blocking the active sites. The total pore volume of the Co-MoS_2_/Al_2_O_3_ catalyst was reduced by 1/3 due to carbon deposition in the initial stage (Fonseca et al., 1996). In our previous works, the Raman characterization of spent Co/MoS_2_ catalysts after running for 72 h at 220°C showed typical peaks at 1,350 and 1,586 cm^−1^ attributed to graphitic carbon species. In addition, the coke content was 4.4 wt.% determined by CHNS analysis, and the combination of carbon deposition and sulfur loss jointly resulted in the decrease of HDO conversion by 2.8% (Zhang C. et al., 2021). In order to reduce the formation of coking, some measures could be adopted such as reducing the acidity of catalysts or increasing the hydrogen pressure.

## Conclusion and Future Perspectives

In this mini-review, we summarized the recent progress of unsupported MoS_2_-based catalysts in the HDO of bio-oil. Reducing the layer number of MoS_2_, especially to a single-layer could significantly improve the HDO activity. Creating defects within the basal planes is also an effective way to enhance the HDO activity, especially for metal-promoted MoS_2_ catalysts because it provides additional anchoring sites. The regulation of HDO selectivity is easily realized by just adjusting the layer number of MoS_2_. Co and Pt doping can both significantly improve the HDO activity and stability of MoS_2_ catalysts, while Co-promoted catalysts are more potential due to the minimized H_2_ consumption. Four different active phases including Co-Mo-S, CoS_2_-MoS_2_, Co-S-Mo, and Co NPs-MoS_2_ have been proposed to construct highly efficient CoMo bimetallic catalysts. Sulfur loss and coking are the main factors to cause deactivation, which could be alleviated by co-feeding of sulfiding agents, surface hydrophobic treatment, and lowering the reaction temperature.

Although tremendous progress has been achieved in the construction of efficient MoS_2_-based HDO catalysts, there are still some challenges to be solved in future research. 1) Simple synthesis of monolayer MoS_2_ nanosheets with rich defects which are ideal supports to prepare CoMo bimetallic catalysts. 2) Experimental and theoretical comparison of the four different active phases of Co-promoted catalysts to figure out which model is best for designing highly efficient CoMo bimetallic catalysts. 3) Deactivation study by using real bio-oil as feedstock and determination of the sulfur loss and coke content.
